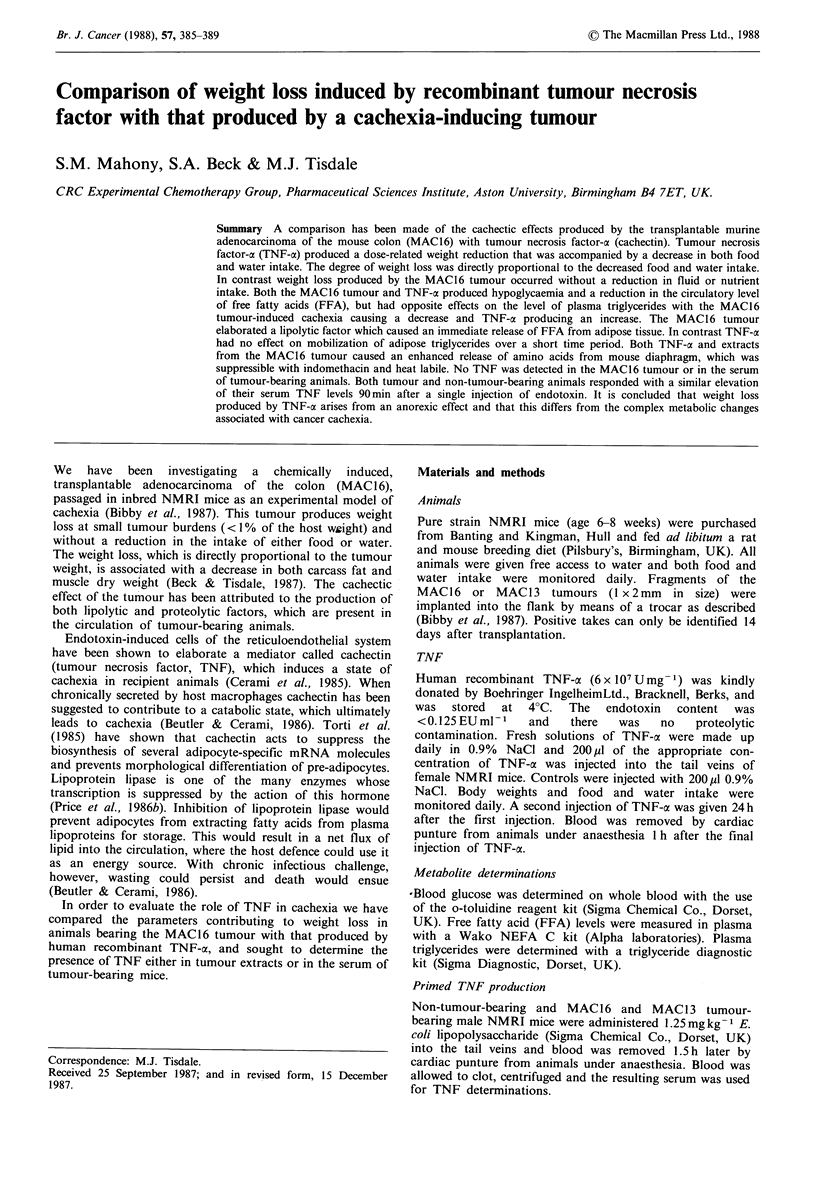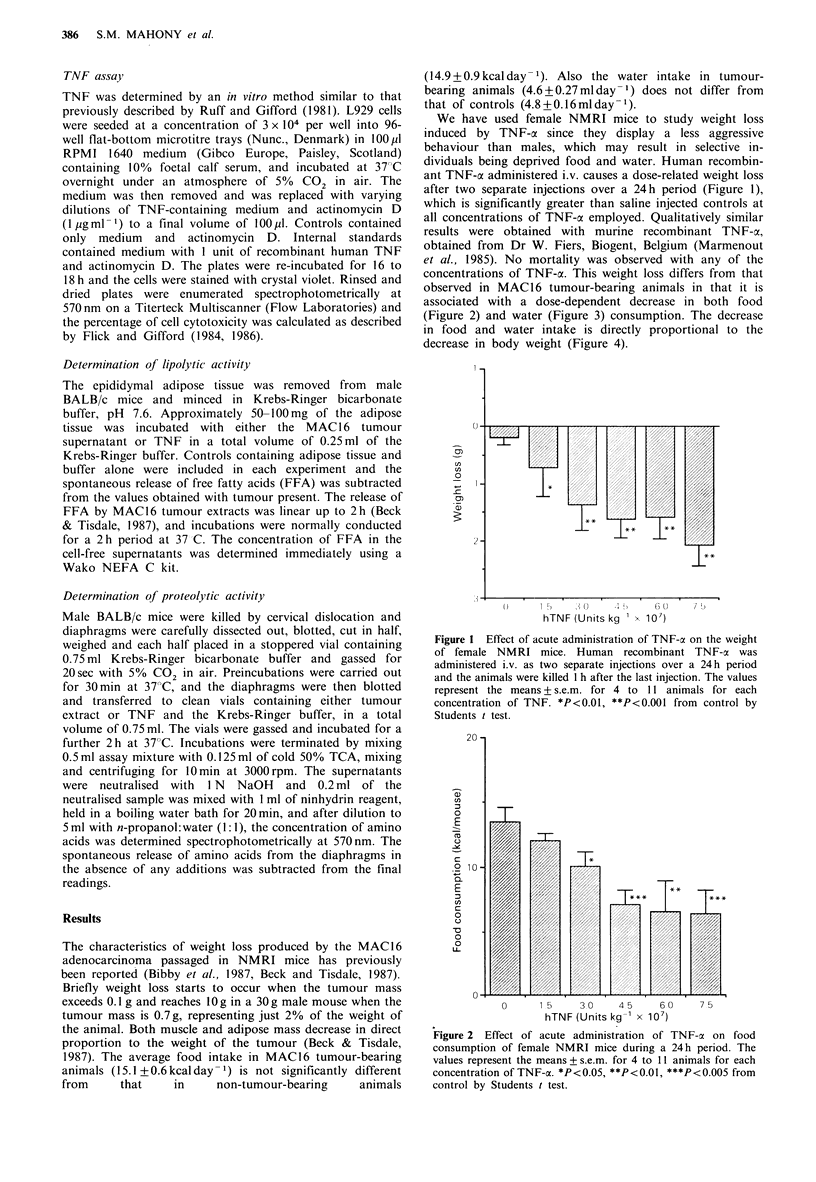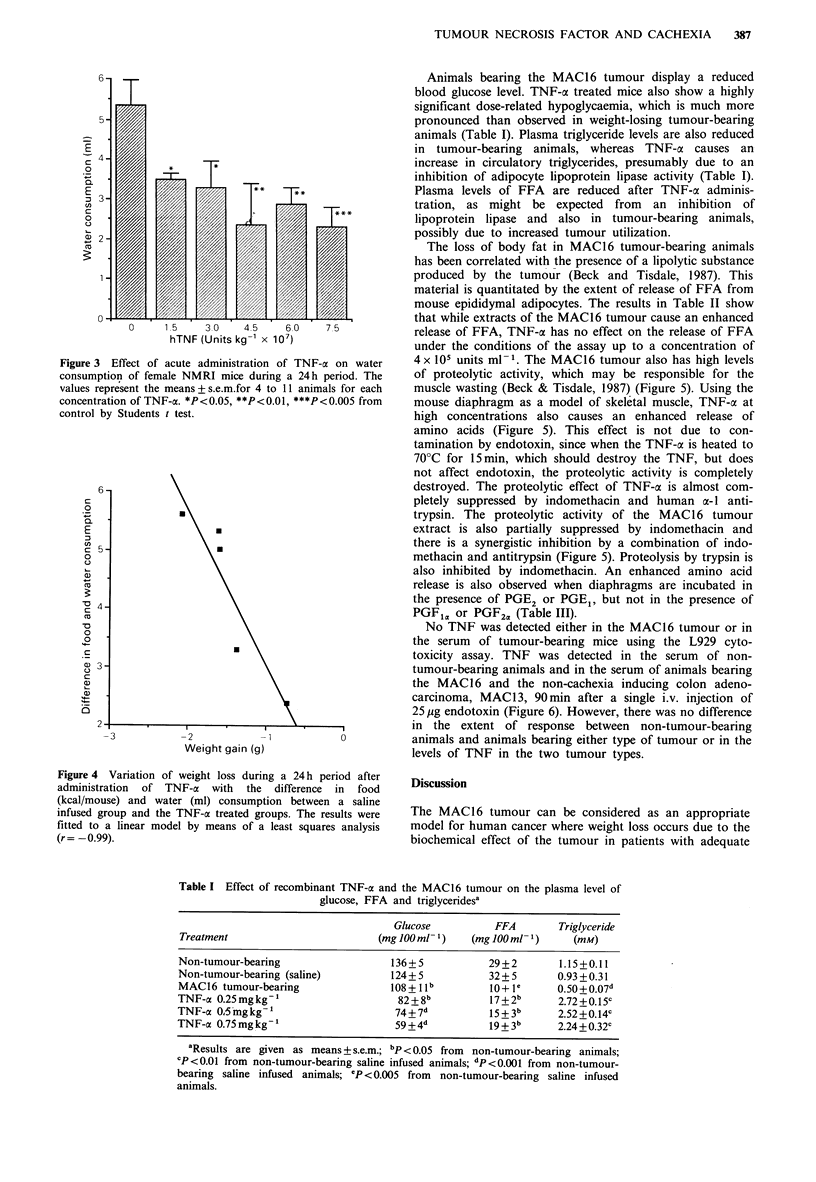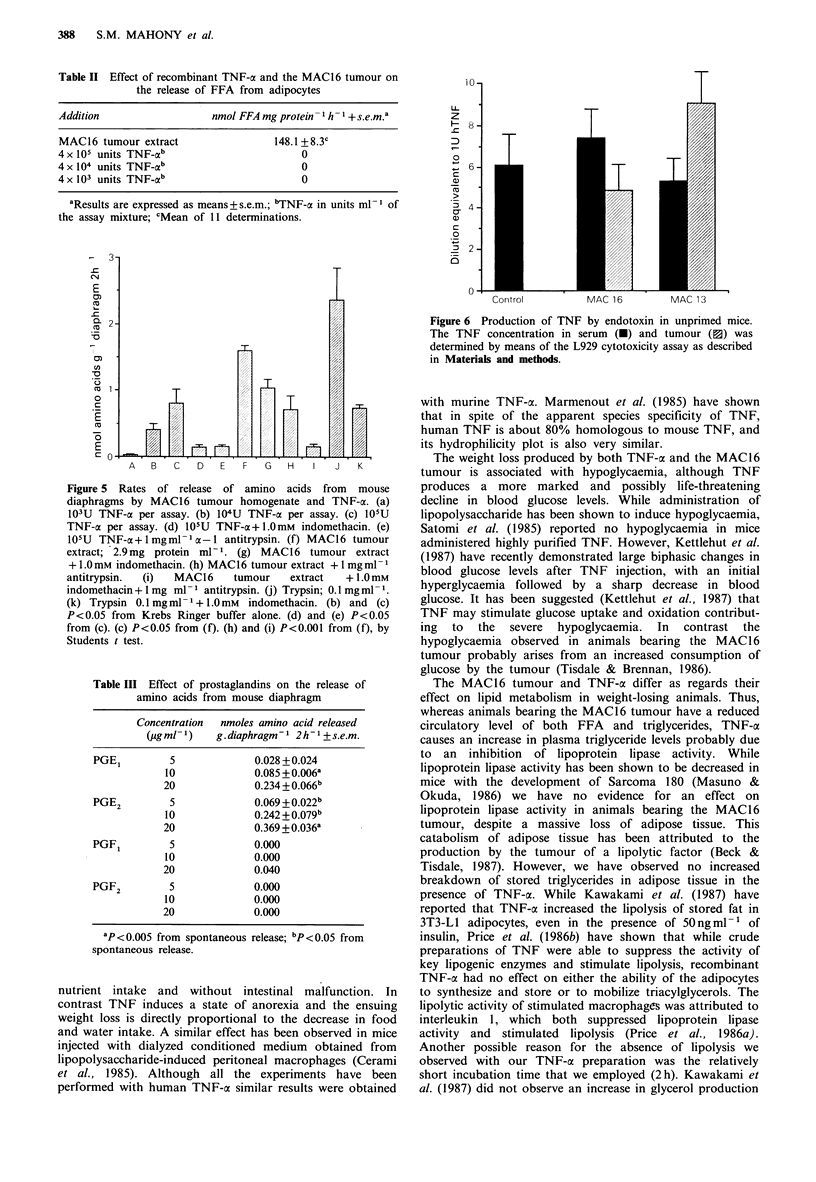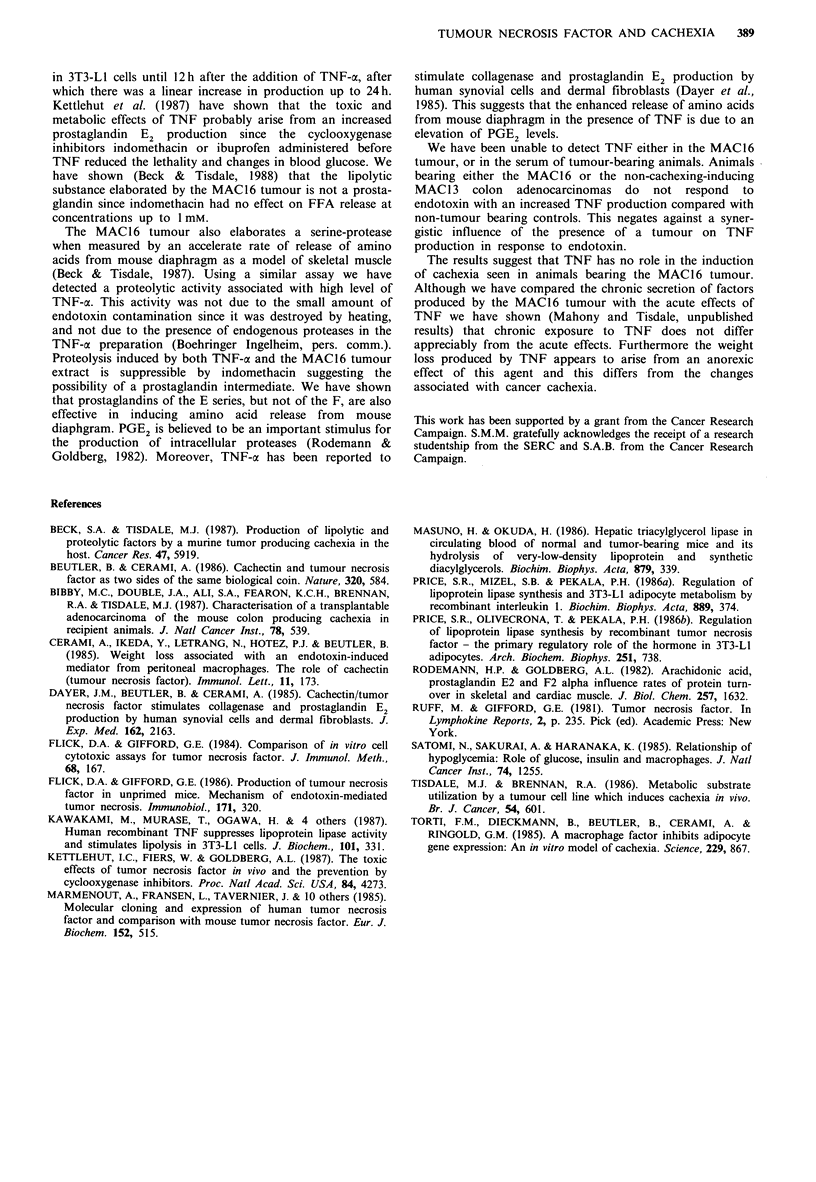# Comparison of weight loss induced by recombinant tumour necrosis factor with that produced by a cachexia-inducing tumour.

**DOI:** 10.1038/bjc.1988.87

**Published:** 1988-04

**Authors:** S. M. Mahony, S. A. Beck, M. J. Tisdale

**Affiliations:** CRC Experimental Chemotherapy Group, Pharmaceutical Sciences Institute, Aston University, Birmingham, UK.

## Abstract

A comparison has been made of the cachectic effects produced by the transplantable murine adenocarcinoma of the mouse colon (MAC16) with tumour necrosis factor-alpha (cachectin). Tumour necrosis factor-alpha (TNF-alpha) produced a dose-related weight reduction that was accompanied by a decrease in both food and water intake. The degree of weight loss was directly proportional to the decreased food and water intake. In contrast weight loss produced by the MAC16 tumour occurred without a reduction in fluid or nutrient intake. Both the MAC16 tumour and TNF-alpha produced hypoglycaemia and a reduction in the circulatory level of free fatty acids (FFA), but had opposite effects on the level of plasma triglycerides with the MAC16 tumour-induced cachexia causing a decrease and TNF-alpha producing an increase. The MAC16 tumour elaborated a lipolytic factor which caused an immediate release of FFA from adipose tissue. In contrast TNF-alpha had no effect on mobilization of adipose triglycerides over a short time period. Both TNF-alpha and extracts from the MAC16 tumour caused an enhanced release of amino acids from mouse diaphragm, which was suppressible with indomethacin and heat labile. No TNF was detected in the MAC16 tumour or in the serum of tumour-bearing animals. Both tumour and non-tumour-bearing animals responded with a similar elevation of their serum TNF levels 90 min after a single injection of endotoxin. It is concluded that weight loss produced by TNF-alpha arises from an anorexic effect and that this differs from the complex metabolic changes associated with cancer cachexia.


Br. J. Cancer (1988), 57, 385-389 © The Macmillan Press Ltd., 1988

### Comparison of weight loss induced by recombinant tumour necrosis factor with that produced by a cachexia-inducing tumour

S.M. Mahony, S.A. Beck & M.J. Tisdale

*CRC Experimental Chemotherapy Group, Pharmaceutical Sciences Institute, Aston University, Birmingham B4 7ET, UK.*

**Summary** A comparison has been made of the cachectic effects produced by the transplantable murine adenocarcinoma of the mouse colon (MAC16) with tumour necrosis factor-α (cachectin). Tumour necrosis factor-α (TNF-α) produced a dose-related weight reduction that was accompanied by a decrease in both food and water intake. The degree of weight loss was directly proportional to the decreased food and water intake. In contrast weight loss produced by the MAC16 tumour occurred without a reduction in fluid or nutrient intake. Both the MAC16 tumour and TNF-α produced hypoglycaemia and a reduction in the circulatory level of free fatty acids (FFA), but had opposite effects on the level of plasma triglycerides with the MAC16 tumour-induced cachexia causing a decrease and TNF-α producing an increase. The MAC16 tumour elaborated a lipolytic factor which caused an immediate release of FFA from adipose tissue. In contrast TNF-α had no effect on mobilization of adipose triglycerides over a short time period. Both TNF-α and extracts from the MAC16 tumour caused an enhanced release of amino acids from mouse diaphragm, which was suppressible with indomethacin and heat labile. No TNF was detected in the MAC16 tumour or in the serum of tumour-bearing animals. Both tumour and non-tumour-bearing animals responded with a similar elevation of their serum TNF levels 90 min after a single injection of endotoxin. It is concluded that weight loss produced by TNF-α arises from an anorexic effect and that this differs from the complex metabolic changes associated with cancer cachexia.

We have been investigating a chemically induced, transplantable adenocarcinoma of the colon (MAC16), passaged in inbred NMRI mice as an experimental model of cachexia (Bibby *et al.*, 1987). This tumour produces weight loss at small tumour burdens (<1% of the host weight) and without a reduction in the intake of either food or water. The weight loss, which is directly proportional to the tumour weight, is associated with a decrease in both carcass fat and muscle dry weight (Beck & Tisdale, 1987). The cachectic effect of the tumour has been attributed to the production of both lipolytic and proteolytic factors, which are present in the circulation of tumour-bearing animals.

Endotoxin-induced cells of the reticuloendothelial system have been shown to elaborate a mediator called cachectin (tumour necrosis factor, TNF), which induces a state of cachexia in recipient animals (Cerami *et al.*, 1985). When chronically secreted by host macrophages cachectin has been suggested to contribute to a catabolic state, which ultimately leads to cachexia (Beutler & Cerami, 1986). Torti *et al.* (1985) have shown that cachectin acts to suppress the biosynthesis of several adipocyte-specific mRNA molecules and prevents morphological differentiation of pre-adipocytes. Lipoprotein lipase is one of the many enzymes whose transcription is suppressed by the action of this hormone (Price *et al.*, 1986*b*). Inhibition of lipoprotein lipase would prevent adipocytes from extracting fatty acids from plasma lipoproteins for storage. This would result in a net flux of lipid into the circulation, where the host defence could use it as an energy source. With chronic infectious challenge, however, wasting could persist and death would ensue (Beutler & Cerami, 1986).

In order to evaluate the role of TNF in cachexia we have compared the parameters contributing to weight loss in animals bearing the MAC16 tumour with that produced by human recombinant TNF-α, and sought to determine the presence of TNF either in tumour extracts or in the serum of tumour-bearing mice.

Correspondence: M.J. Tisdale.
Received 25 September 1987; and in revised form, 15 December 1987.

### Materials and methods

### *Animals*

Pure strain NMRI mice (age 6–8 weeks) were purchased from Banting and Kingman, Hull and fed *ad libitum* a rat and mouse breeding diet (Pilsbury's, Birmingham, UK). All animals were given free access to water and both food and water intake were monitored daily. Fragments of the MAC16 or MAC13 tumours (1×2mm in size) were implanted into the flank by means of a trocar as described (Bibby *et al.*, 1987). Positive takes can only be identified 14 days after transplantation.

### *TNF*

Human recombinant TNF-α ($6\times10^7\,U\,mg^{-1}$) was kindly donated by Boehringer IngelheimLtd., Bracknell, Berks, and was stored at 4°C. The endotoxin content was $<0.125\,EU\,ml^{-1}$ and there was no proteolytic contamination. Fresh solutions of TNF-α were made up daily in 0.9% NaCl and 200 μl of the appropriate concentration of TNF-α was injected into the tail veins of female NMRI mice. Controls were injected with 200 μl 0.9% NaCl. Body weights and food and water intake were monitored daily. A second injection of TNF-α was given 24 h after the first injection. Blood was removed by cardiac punture from animals under anaesthesia 1 h after the final injection of TNF-α.

### *Metabolite determinations*

Blood glucose was determined on whole blood with the use of the o-toluidine reagent kit (Sigma Chemical Co., Dorset, UK). Free fatty acid (FFA) levels were measured in plasma with a Wako NEFA C kit (Alpha laboratories). Plasma triglycerides were determined with a triglyceride diagnostic kit (Sigma Diagnostic, Dorset, UK).

### *Primed TNF production*

Non-tumour-bearing and MAC16 and MAC13 tumour-bearing male NMRI mice were administered $1.25\,mg\,kg^{-1}$ *E. coli* lipopolysaccharide (Sigma Chemical Co., Dorset, UK) into the tail veins and blood was removed 1.5 h later by cardiac punture from animals under anaesthesia. Blood was allowed to clot, centrifuged and the resulting serum was used for TNF determinations.

*TNF assay*

TNF was determined by an *in vitro* method similar to that previously described by Ruff and Gifford (1981). L929 cells were seeded at a concentration of $3 \times 10^4$ per well into 96-well flat-bottom microtitre trays (Nunc., Denmark) in 100 $\mu$l RPMI 1640 medium (Gibco Europe, Paisley, Scotland) containing 10% foetal calf serum, and incubated at 37°C overnight under an atmosphere of 5% $CO_2$ in air. The medium was then removed and was replaced with varying dilutions of TNF-containing medium and actinomycin D (1 $\mu g\, ml^{-1}$) to a final volume of 100 $\mu$l. Controls contained only medium and actinomycin D. Internal standards contained medium with 1 unit of recombinant human TNF and actinomycin D. The plates were re-incubated for 16 to 18 h and the cells were stained with crystal violet. Rinsed and dried plates were enumerated spectrophotometrically at 570 nm on a Titerteck Multiscanner (Flow Laboratories) and the percentage of cell cytotoxicity was calculated as described by Flick and Gifford (1984, 1986).

*Determination of lipolytic activity*

The epididymal adipose tissue was removed from male BALB/c mice and minced in Krebs-Ringer bicarbonate buffer, pH 7.6. Approximately 50–100 mg of the adipose tissue was incubated with either the MAC16 tumour supernatant or TNF in a total volume of 0.25 ml of the Krebs-Ringer buffer. Controls containing adipose tissue and buffer alone were included in each experiment and the spontaneous release of free fatty acids (FFA) was subtracted from the values obtained with tumour present. The release of FFA by MAC16 tumour extracts was linear up to 2 h (Beck & Tisdale, 1987), and incubations were normally conducted for a 2 h period at 37°C. The concentration of FFA in the cell-free supernatants was determined immediately using a Wako NEFA C kit.

*Determination of proteolytic activity*

Male BALB/c mice were killed by cervical dislocation and diaphragms were carefully dissected out, blotted, cut in half, weighed and each half placed in a stoppered vial containing 0.75 ml Krebs-Ringer bicarbonate buffer and gassed for 20 sec with 5% $CO_2$ in air. Preincubations were carried out for 30 min at 37°C, and the diaphragms were then blotted and transferred to clean vials containing either tumour extract or TNF and the Krebs-Ringer buffer, in a total volume of 0.75 ml. The vials were gassed and incubated for a further 2 h at 37°C. Incubations were terminated by mixing 0.5 ml assay mixture with 0.125 ml of cold 50% TCA, mixing and centrifuging for 10 min at 3000 rpm. The supernatants were neutralised with 1 N NaOH and 0.2 ml of the neutralised sample was mixed with 1 ml of ninhydrin reagent, held in a boiling water bath for 20 min, and after dilution to 5 ml with *n*-propanol:water (1:1), the concentration of amino acids was determined spectrophotometrically at 570 nm. The spontaneous release of amino acids from the diaphragms in the absence of any additions was subtracted from the final readings.

### Results

The characteristics of weight loss produced by the MAC16 adenocarcinoma passaged in NMRI mice has previously been reported (Bibby *et al.*, 1987, Beck and Tisdale, 1987). Briefly weight loss starts to occur when the tumour mass exceeds 0.1 g and reaches 10 g in a 30 g male mouse when the tumour mass is 0.7 g, representing just 2% of the weight of the animal. Both muscle and adipose mass decrease in direct proportion to the weight of the tumour (Beck & Tisdale, 1987). The average food intake in MAC16 tumour-bearing animals ($15.1 \pm 0.6$ kcal day$^{-1}$) is not significantly different from that in non-tumour-bearing animals ($14.9 \pm 0.9$ kcal day$^{-1}$). Also the water intake in tumour-bearing animals ($4.6 \pm 0.27$ ml day$^{-1}$) does not differ from that of controls ($4.8 \pm 0.16$ ml day$^{-1}$).

We have used female NMRI mice to study weight loss induced by TNF-α since they display a less aggressive behaviour than males, which may result in selective individuals being deprived food and water. Human recombinant TNF-α administered i.v. causes a dose-related weight loss after two separate injections over a 24 h period (Figure 1), which is significantly greater than saline injected controls at all concentrations of TNF-α employed. Qualitatively similar results were obtained with murine recombinant TNF-α, obtained from Dr W. Fiers, Biogent, Belgium (Marmenout *et al.*, 1985). No mortality was observed with any of the concentrations of TNF-α. This weight loss differs from that observed in MAC16 tumour-bearing animals in that it is associated with a dose-dependent decrease in both food (Figure 2) and water (Figure 3) consumption. The decrease in food and water intake is directly proportional to the decrease in body weight (Figure 4).

**Figure 1** Effect of acute administration of TNF-α on the weight of female NMRI mice. Human recombinant TNF-α was administered i.v. as two separate injections over a 24 h period and the animals were killed 1 h after the last injection. The values represent the means ± s.e.m. for 4 to 11 animals for each concentration of TNF. *$P < 0.01$, **$P < 0.001$ from control by Students *t* test.

**Figure 2** Effect of acute administration of TNF-α on food consumption of female NMRI mice during a 24 h period. The values represent the means ± s.e.m. for 4 to 11 animals for each concentration of TNF-α. *$P < 0.05$, **$P < 0.01$, ***$P < 0.005$ from control by Students *t* test.

**Figure 3** Effect of acute administration of TNF-$\alpha$ on water consumption of female NMRI mice during a 24 h period. The values represent the means ± s.e.m.for 4 to 11 animals for each concentration of TNF-$\alpha$. $^{*}P<0.05$, $^{**}P<0.01$, $^{***}P<0.005$ from control by Students $t$ test.

**Figure 4** Variation of weight loss during a 24 h period after administration of TNF-$\alpha$ with the difference in food (kcal/mouse) and water (ml) consumption between a saline infused group and the TNF-$\alpha$ treated groups. The results were fitted to a linear model by means of a least squares analysis ($r=-0.99$).

Animals bearing the MAC16 tumour display a reduced blood glucose level. TNF-$\alpha$ treated mice also show a highly significant dose-related hypoglycaemia, which is much more pronounced than observed in weight-losing tumour-bearing animals (Table I). Plasma triglyceride levels are also reduced in tumour-bearing animals, whereas TNF-$\alpha$ causes an increase in circulatory triglycerides, presumably due to an inhibition of adipocyte lipoprotein lipase activity (Table I). Plasma levels of FFA are reduced after TNF-$\alpha$ administration, as might be expected from an inhibition of lipoprotein lipase and also in tumour-bearing animals, possibly due to increased tumour utilization.

The loss of body fat in MAC16 tumour-bearing animals has been correlated with the presence of a lipolytic substance produced by the tumour (Beck and Tisdale, 1987). This material is quantitated by the extent of release of FFA from mouse epididymal adipocytes. The results in Table II show that while extracts of the MAC16 tumour cause an enhanced release of FFA, TNF-$\alpha$ has no effect on the release of FFA under the conditions of the assay up to a concentration of $4\times10^5$ units $ml^{-1}$. The MAC16 tumour also has high levels of proteolytic activity, which may be responsible for the muscle wasting (Beck & Tisdale, 1987) (Figure 5). Using the mouse diaphragm as a model of skeletal muscle, TNF-$\alpha$ at high concentrations also causes an enhanced release of amino acids (Figure 5). This effect is not due to contamination by endotoxin, since when the TNF-$\alpha$ is heated to 70°C for 15 min, which should destroy the TNF, but does not affect endotoxin, the proteolytic activity is completely destroyed. The proteolytic effect of TNF-$\alpha$ is almost completely suppressed by indomethacin and human $\alpha$-1 antitrypsin. The proteolytic activity of the MAC16 tumour extract is also partially suppressed by indomethacin and there is a synergistic inhibition by a combination of indomethacin and antitrypsin (Figure 5). Proteolysis by trypsin is also inhibited by indomethacin. An enhanced amino acid release is also observed when diaphragms are incubated in the presence of $PGE_2$ or $PGE_1$, but not in the presence of $PGF_{1\alpha}$ or $PGF_{2\alpha}$ (Table III).

No TNF was detected either in the MAC16 tumour or in the serum of tumour-bearing mice using the L929 cytotoxicity assay. TNF was detected in the serum of non-tumour-bearing animals and in the serum of animals bearing the MAC16 and the non-cachexia inducing colon adenocarcinoma, MAC13, 90 min after a single i.v. injection of 25 $\mu$g endotoxin (Figure 6). However, there was no difference in the extent of response between non-tumour-bearing animals and animals bearing either type of tumour or in the levels of TNF in the two tumour types.

### Discussion

The MAC16 tumour can be considered as an appropriate model for human cancer where weight loss occurs due to the biochemical effect of the tumour in patients with adequate

**Table I** Effect of recombinant TNF-$\alpha$ and the MAC16 tumour on the plasma level of glucose, FFA and triglycerides[a]

| *Treatment* | *Glucose* (*mg 100 ml*$^{-1}$) | *FFA* (*mg 100 ml*$^{-1}$) | *Triglyceride* (*mM*) |
|---|---|---|---|
| Non-tumour-bearing | 136±5 | 29±2 | 1.15±0.11 |
| Non-tumour-bearing (saline) | 124±5 | 32±5 | 0.93±0.31 |
| MAC16 tumour-bearing | 108±11[b] | 10+1[e] | 0.50±0.07[d] |
| TNF-$\alpha$ 0.25 mg kg$^{-1}$ | 82±8[b] | 17±2[b] | 2.72±0.15[c] |
| TNF-$\alpha$ 0.5 mg kg$^{-1}$ | 74±7[d] | 15±3[b] | 2.52±0.14[c] |
| TNF-$\alpha$ 0.75 mg kg$^{-1}$ | 59±4[d] | 19±3[b] | 2.24±0.32[c] |

[a]Results are given as means ± s.e.m.; [b]$P<0.05$ from non-tumour-bearing animals; [c]$P<0.01$ from non-tumour-bearing saline infused animals; [d]$P<0.001$ from non-tumour-bearing saline infused animals; [e]$P<0.005$ from non-tumour-bearing saline infused animals.

**Table II** Effect of recombinant TNF-α and the MAC16 tumour on the release of FFA from adipocytes

| *Addition* | *nmol FFA mg protein*$^{-1}$ *h*$^{-1}$ + *s.e.m.*[a] |
|---|---|
| MAC16 tumour extract | 148.1 ± 8.3[c] |
| $4 \times 10^5$ units TNF-α[b] | 0 |
| $4 \times 10^4$ units TNF-α[b] | 0 |
| $4 \times 10^3$ units TNF-α[b] | 0 |

[a]Results are expressed as means ± s.e.m.; [b]TNF-α in units ml$^{-1}$ of the assay mixture; [c]Mean of 11 determinations.

**Figure 5** Rates of release of amino acids from mouse diaphragms by MAC16 tumour homogenate and TNF-α. (a) $10^3$U TNF-α per assay. (b) $10^4$U TNF-α per assay. (c) $10^5$U TNF-α per assay. (d) $10^5$U TNF-α + 1.0 mM indomethacin. (e) $10^5$U TNF-α + 1 mg ml$^{-1}$ α − 1 antitrypsin. (f) MAC16 tumour extract; 2.9 mg protein ml$^{-1}$. (g) MAC16 tumour extract + 1.0 mM indomethacin. (h) MAC16 tumour extract + 1 mg ml$^{-1}$ antitrypsin. (i) MAC16 tumour extract + 1.0 mM indomethacin + 1 mg ml$^{-1}$ antitrypsin. (j) Trypsin; 0.1 mg ml$^{-1}$. (k) Trypsin 0.1 mg ml$^{-1}$ + 1.0 mM indomethacin. (b) and (c) $P < 0.05$ from Krebs Ringer buffer alone. (d) and (e) $P < 0.05$ from (c). (c) $P < 0.05$ from (f). (h) and (i) $P < 0.001$ from (f), by Students $t$ test.

**Table III** Effect of prostaglandins on the release of amino acids from mouse diaphragm

| | *Concentration* (*μg ml*$^{-1}$) | *nmoles amino acid released g.diaphragm*$^{-1}$ *2 h*$^{-1}$ ± *s.e.m.* |
|---|---|---|
| $PGE_1$ | 5 | 0.028 ± 0.024 |
| | 10 | 0.085 ± 0.006[a] |
| | 20 | 0.234 ± 0.066[b] |
| $PGE_2$ | 5 | 0.069 ± 0.022[b] |
| | 10 | 0.242 ± 0.079[b] |
| | 20 | 0.369 ± 0.036[a] |
| $PGF_1$ | 5 | 0.000 |
| | 10 | 0.000 |
| | 20 | 0.040 |
| $PGF_2$ | 5 | 0.000 |
| | 10 | 0.000 |
| | 20 | 0.000 |

[a]$P < 0.005$ from spontaneous release; [b]$P < 0.05$ from spontaneous release.

nutrient intake and without intestinal malfunction. In contrast TNF induces a state of anorexia and the ensuing weight loss is directly proportional to the decrease in food and water intake. A similar effect has been observed in mice injected with dialyzed conditioned medium obtained from lipopolysaccharide-induced peritoneal macrophages (Cerami *et al.*, 1985). Although all the experiments have been performed with human TNF-α similar results were obtained

**Figure 6** Production of TNF by endotoxin in unprimed mice. The TNF concentration in serum (■) and tumour (▨) was determined by means of the L929 cytotoxicity assay as described in **Materials and methods**.

with murine TNF-α. Marmenout *et al.* (1985) have shown that in spite of the apparent species specificity of TNF, human TNF is about 80% homologous to mouse TNF, and its hydrophilicity plot is also very similar.

The weight loss produced by both TNF-α and the MAC16 tumour is associated with hypoglycaemia, although TNF produces a more marked and possibly life-threatening decline in blood glucose levels. While administration of lipopolysaccharide has been shown to induce hypoglycaemia, Satomi *et al.* (1985) reported no hypoglycaemia in mice administered highly purified TNF. However, Kettlehut *et al.* (1987) have recently demonstrated large biphasic changes in blood glucose levels after TNF injection, with an initial hyperglycaemia followed by a sharp decrease in blood glucose. It has been suggested (Kettlehut *et al.*, 1987) that TNF may stimulate glucose uptake and oxidation contributing to the severe hypoglycaemia. In contrast the hypoglycaemia observed in animals bearing the MAC16 tumour probably arises from an increased consumption of glucose by the tumour (Tisdale & Brennan, 1986).

The MAC16 tumour and TNF-α differ as regards their effect on lipid metabolism in weight-losing animals. Thus, whereas animals bearing the MAC16 tumour have a reduced circulatory level of both FFA and triglycerides, TNF-α causes an increase in plasma triglyceride levels probably due to an inhibition of lipoprotein lipase activity. While lipoprotein lipase activity has been shown to be decreased in mice with the development of Sarcoma 180 (Masuno & Okuda, 1986) we have no evidence for an effect on lipoprotein lipase activity in animals bearing the MAC16 tumour, despite a massive loss of adipose tissue. This catabolism of adipose tissue has been attributed to the production by the tumour of a lipolytic factor (Beck & Tisdale, 1987). However, we have observed no increased breakdown of stored triglycerides in adipose tissue in the presence of TNF-α. While Kawakami *et al.* (1987) have reported that TNF-α increased the lipolysis of stored fat in 3T3-L1 adipocytes, even in the presence of 50 ng ml$^{-1}$ of insulin, Price *et al.* (1986*b*) have shown that while crude preparations of TNF were able to suppress the activity of key lipogenic enzymes and stimulate lipolysis, recombinant TNF-α had no effect on either the ability of the adipocytes to synthesize and store or to mobilize triacylglycerols. The lipolytic activity of stimulated macrophages was attributed to interleukin 1, which both suppressed lipoprotein lipase activity and stimulated lipolysis (Price *et al.*, 1986*a*). Another possible reason for the absence of lipolysis we observed with our TNF-α preparation was the relatively short incubation time that we employed (2 h). Kawakami *et al.* (1987) did not observe an increase in glycerol production in 3T3-L1 cells until 12 h after the addition of TNF-α, after which there was a linear increase in production up to 24 h. Kettlehut *et al.* (1987) have shown that the toxic and metabolic effects of TNF probably arise from an increased prostaglandin $E_2$ production since the cyclooxygenase inhibitors indomethacin or ibuprofen administered before TNF reduced the lethality and changes in blood glucose. We have shown (Beck & Tisdale, 1988) that the lipolytic substance elaborated by the MAC16 tumour is not a prostaglandin since indomethacin had no effect on FFA release at concentrations up to 1 mM.

The MAC16 tumour also elaborates a serine-protease when measured by an accelerate rate of release of amino acids from mouse diaphragm as a model of skeletal muscle (Beck & Tisdale, 1987). Using a similar assay we have detected a proteolytic activity associated with high level of TNF-α. This activity was not due to the small amount of endotoxin contamination since it was destroyed by heating, and not due to the presence of endogenous proteases in the TNF-α preparation (Boehringer Ingelheim, pers. comm.). Proteolysis induced by both TNF-α and the MAC16 tumour extract is suppressible by indomethacin suggesting the possibility of a prostaglandin intermediate. We have shown that prostaglandins of the E series, but not of the F, are also effective in inducing amino acid release from mouse diaphgram. $PGE_2$ is believed to be an important stimulus for the production of intracellular proteases (Rodemann & Goldberg, 1982). Moreover, TNF-α has been reported to stimulate collagenase and prostaglandin $E_2$ production by human synovial cells and dermal fibroblasts (Dayer *et al.*, 1985). This suggests that the enhanced release of amino acids from mouse diaphragm in the presence of TNF is due to an elevation of $PGE_2$ levels.

We have been unable to detect TNF either in the MAC16 tumour, or in the serum of tumour-bearing animals. Animals bearing either the MAC16 or the non-cachexing-inducing MAC13 colon adenocarcinomas do not respond to endotoxin with an increased TNF production compared with non-tumour bearing controls. This negates against a synergistic influence of the presence of a tumour on TNF production in response to endotoxin.

The results suggest that TNF has no role in the induction of cachexia seen in animals bearing the MAC16 tumour. Although we have compared the chronic secretion of factors produced by the MAC16 tumour with the acute effects of TNF we have shown (Mahony and Tisdale, unpublished results) that chronic exposure to TNF does not differ appreciably from the acute effects. Furthermore the weight loss produced by TNF appears to arise from an anorexic effect of this agent and this differs from the changes associated with cancer cachexia.

This work has been supported by a grant from the Cancer Research Campaign. S.M.M. gratefully acknowledges the receipt of a research studentship from the SERC and S.A.B. from the Cancer Research Campaign.

### References

BECK, S.A. & TISDALE, M.J. (1987). Production of lipolytic and proteolytic factors by a murine tumor producing cachexia in the host. *Cancer Res.* **47**, 5919.

BEUTLER, B. & CERAMI, A. (1986). Cachectin and tumour necrosis factor as two sides of the same biological coin. *Nature*, **320**, 584.

BIBBY, M.C., DOUBLE, J.A., ALI, S.A., FEARON, K.C.H., BRENNAN, R.A. & TISDALE, M.J. (1987). Characterisation of a transplantable adenocarcinoma of the mouse colon producing cachexia in recipient animals. *J. Natl Cancer Inst.*, **78**, 539.

CERAMI, A., IKEDA, Y., LETRANG, N., HOTEZ, P.J. & BEUTLER, B. (1985). Weight loss associated with an endotoxin-induced mediator from peritoneal macrophages. The role of cachectin (tumour necrosis factor). *Immunol. Lett.*, **11**, 173.

DAYER, J.M., BEUTLER, B. & CERAMI, A. (1985). Cachectin/tumor necrosis factor stimulates collagenase and prostaglandin $E_2$ production by human synovial cells and dermal fibroblasts. *J. Exp. Med.* **162**, 2163.

FLICK, D.A. & GIFFORD, G.E. (1984). Comparison of *in vitro* cell cytotoxic assays for tumor necrosis factor. *J. Immunol. Meth.*, **68**, 167.

FLICK, D.A. & GIFFORD, G.E. (1986). Production of tumour necrosis factor in unprimed mice. Mechanism of endotoxin-mediated tumor necrosis. *Immunobiol.*, **171**, 320.

KAWAKAMI, M., MURASE, T., OGAWA, H. & 4 others (1987). Human recombinant TNF suppresses lipoprotein lipase activity and stimulates lipolysis in 3T3-L1 cells. *J. Biochem.*, **101**, 331.

KETTLEHUT, I.C., FIERS, W. & GOLDBERG, A.L. (1987). The toxic effects of tumor necrosis factor *in vivo* and the prevention by cyclooxygenase inhibitors. *Proc. Natl Acad. Sci. USA*, **84**, 4273.

MARMENOUT, A., FRANSEN, L., TAVERNIER, J. & 10 others (1985). Molecular cloning and expression of human tumor necrosis factor and comparison with mouse tumor necrosis factor. *Eur. J. Biochem.* **152**, 515.

MASUNO, H. & OKUDA, H. (1986). Hepatic triacylglycerol lipase in circulating blood of normal and tumor-bearing mice and its hydrolysis of very-low-density lipoprotein and synthetic diacylglycerols. *Biochim. Biophys. Acta*, **879**, 339.

PRICE, S.R., MIZEL, S.B. & PEKALA, P.H. (1986*a*). Regulation of lipoprotein lipase synthesis and 3T3-L1 adipocyte metabolism by recombinant interleukin 1. *Biochim. Biophys. Acta*, **889**, 374.

PRICE, S.R., OLIVECRONA, T. & PEKALA, P.H. (1986*b*). Regulation of lipoprotein lipase synthesis by recombinant tumor necrosis factor – the primary regulatory role of the hormone in 3T3-L1 adipocytes. *Arch. Biochem. Biophys.* **251**, 738.

RODEMANN, H.P. & GOLDBERG, A.L. (1982). Arachidonic acid, prostaglandin E2 and F2 alpha influence rates of protein turnover in skeletal and cardiac muscle. *J. Biol. Chem.* **257**, 1632.

RUFF, M. & GIFFORD, G.E. (1981). Tumor necrosis factor. In *Lymphokine Reports*, **2**, p. 235. Pick (ed). Academic Press: New York.

SATOMI, N., SAKURAI, A. & HARANAKA, K. (1985). Relationship of hypoglycemia: Role of glucose, insulin and macrophages. *J. Natl Cancer Inst.*, **74**, 1255.

TISDALE, M.J. & BRENNAN, R.A. (1986). Metabolic substrate utilization by a tumour cell line which induces cachexia *in vivo*. *Br. J. Cancer*, **54**, 601.

TORTI, F.M., DIECKMANN, B., BEUTLER, B., CERAMI, A. & RINGOLD, G.M. (1985). A macrophage factor inhibits adipocyte gene expression: An *in vitro* model of cachexia. *Science*, **229**, 867.